# R. Randal Bollinger, M.D., Ph.D., Master Surgeon

**DOI:** 10.3389/frtra.2024.1469916

**Published:** 2024-08-27

**Authors:** Stuart J. Knechtle, Allan D. Kirk

**Affiliations:** School of Medicine, Duke University, Durham, United States

**Keywords:** transplant, surgeon, research, teacher, mentor, immunology

## Introduction

R. Randal Bollinger began life in Dearborn, Michigan, on 3 October 1944. He graduated in 1966 cum laude from Tulane University, New Orleans, where he majored in biology. During college, he studied biology for a year with Professor Karl Grell in Tubingen, Baden-Württemburg, Germany. He also immersed himself in New Orleans culture, participating as a tuba player in Mardi Gras parades. His affinity for New Orleans and for biological sciences led him to enroll at Tulane University School of Medicine, where he came under the influence of Dr. John MacDonald, a pioneer in kidney transplant surgery. MacDonald and Bollinger published several papers together on the relationship between gut bacterial flora and immune responses in transplantation models (1). At Tulane, Bollinger was elected to the Alpha Omega Alpha honor society and earned a Master of Science in biochemistry. Dr. MacDonald encouraged Bollinger to pursue further scientific training, and Bollinger sought to come to Duke to study immunology with Dr. D. Bernard Amos.

Bernard Amos had been recruited to Duke by the medical school dean Barnes Woodall and surgery department chairman Clarence Gardner because of Amos's immunogenetics background. He had been asked to develop an organ transplant program scientifically based on the then burgeoning immunogenetic principles of histocompatibility, to guide donor–recipient selection. At Duke, Bollinger pursued a Ph.D. in immunology with Amos and Dr. David Scott on the tolerogenic benefits of donor antigens conjugated to recipient spleen cells (2, 3). In addition to his doctoral work at Duke, he was accepted in 1970 into the general surgical residency program of Dr. David C. Sabiston, Jr., in which Bollinger continued to excel. His surgical residency was interrupted by 2 years of service with the United States Airforce. In 1980, Dr. Sabiston asked him to join the Duke surgery faculty, and he was appointed chief of transplantation in 1983.

**Figure 1:**
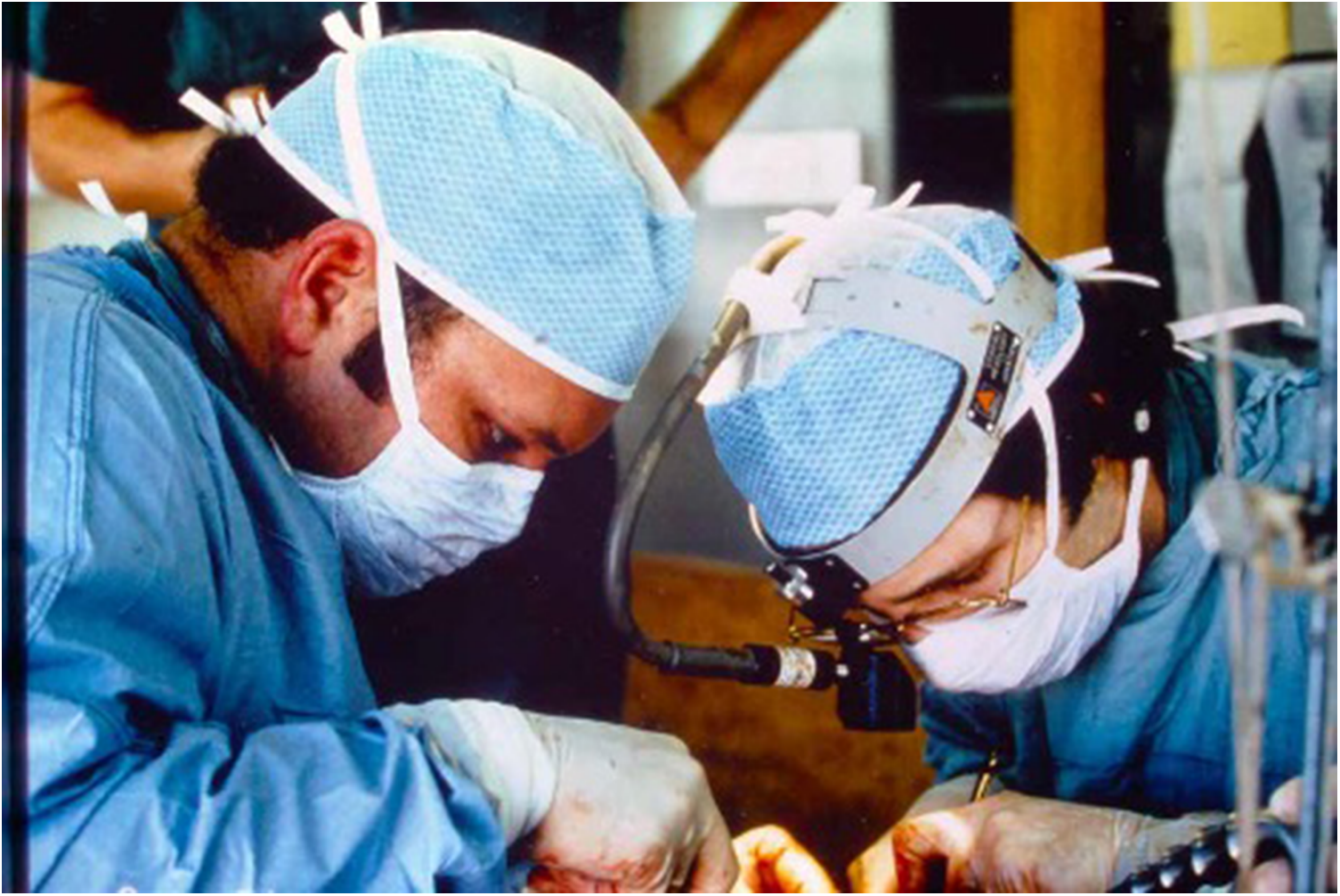
Dr. Randy Bollinger and Dr. Richard McCann performing an experimental liver transplant.

As chief of transplantation at Duke, Dr. Bollinger grew the deceased donor kidney transplant program, as the early years of the program under Drs. Stickel and Seigler had focused on living donor transplants. He also adopted new immunosuppressive agents as they became available in the 1980s, including participating in the clinical trial of OKT3 (anti-CD3T cell-directed monoclonal antibody) and early use of cyclosporine, the first clinically available calcineurin inhibitor. He directed the programmatic development that led to the first liver transplant in North Carolina in 1986, which he personally performed (both the donor and recipient procedures) with the assistance of his colleagues William Meyers and Richard McCann. See Figure 1: Drs. Bollinger and McCann performing experimental liver transplantation. He supported the development of pancreas transplantation by training Dr. Ben Vernon in his lab and recruiting him to the faculty after his fellowship at the University of Wisconsin. Vernon performed the first pancreas transplant at Duke in 1989. In addition to routinely performing demanding organ transplants, Bollinger developed an interest in surgery for inflammatory bowel disease, performing continent ileostomies, or Koch pouches, in combination with total colectomy. He was rapidly promoted to professor of surgery and immunology.

As an administrative leader, in addition to serving as chief of transplantation, Dr. Bollinger served as general surgery division chief, opened a multidisciplinary transplant clinic, and established a Transplant Clinical Business Unit. In 1997, he obtained his MBA from the Fuqua School of Business. His impact was felt in the southeast region as a leader among the North Carolina organ procurement organizations and nationally as a councilor of the American Society of Transplant Surgeons, president of the South-Eastern Organ Procurement Foundation (SEOPF, the nation's first organ procurement body), and president of the United Network of Organ Sharing (UNOS), which evolved from the SEOPF.

His research interests focused on transplant immunology, including immune tolerance, transplantation in sensitized recipients, and xenotransplantation. He received continuous National Institutes of Health funding for over two decades, including a program project in xenotransplantation, and contributed 131 manuscripts to the scientific literature. Major accomplishments included extending pig-to-baboon cardiac xenograft survival using soluble complement factor infusion, transgenic pig hearts expressing human CD59 and decay accelerating factor (DAF) or membrane cofactor protein (MCP) complement regulatory proteins, and the use of *ex vivo* perfusion of porcine livers as a bridge to liver transplantation. He also conducted seminal studies with Stuart Knechtle and Ed Halperin in the use of total lymphoid irradiation as a means of inducing graft acceptance (4).

“Randy,” as he insisted on being called even by his residents, became a beloved mentor, renowned for his patience and good humor in the operating room. His integrity and character were as much revered as his surgical and scientific abilities. His sense of humor was not far beneath his surface, exemplified by his performing a “rain dance” around the operating room table following reperfusion of a transplanted kidney. His dance included musical incantations for rain (urine production) to the percussion accompaniment of a Richardson retractor banging on a sterile stainless-steel bowl. Even if this act failed to elicit urine, it was guaranteed to produce laughter among the operating room team.

**Figure 2:**
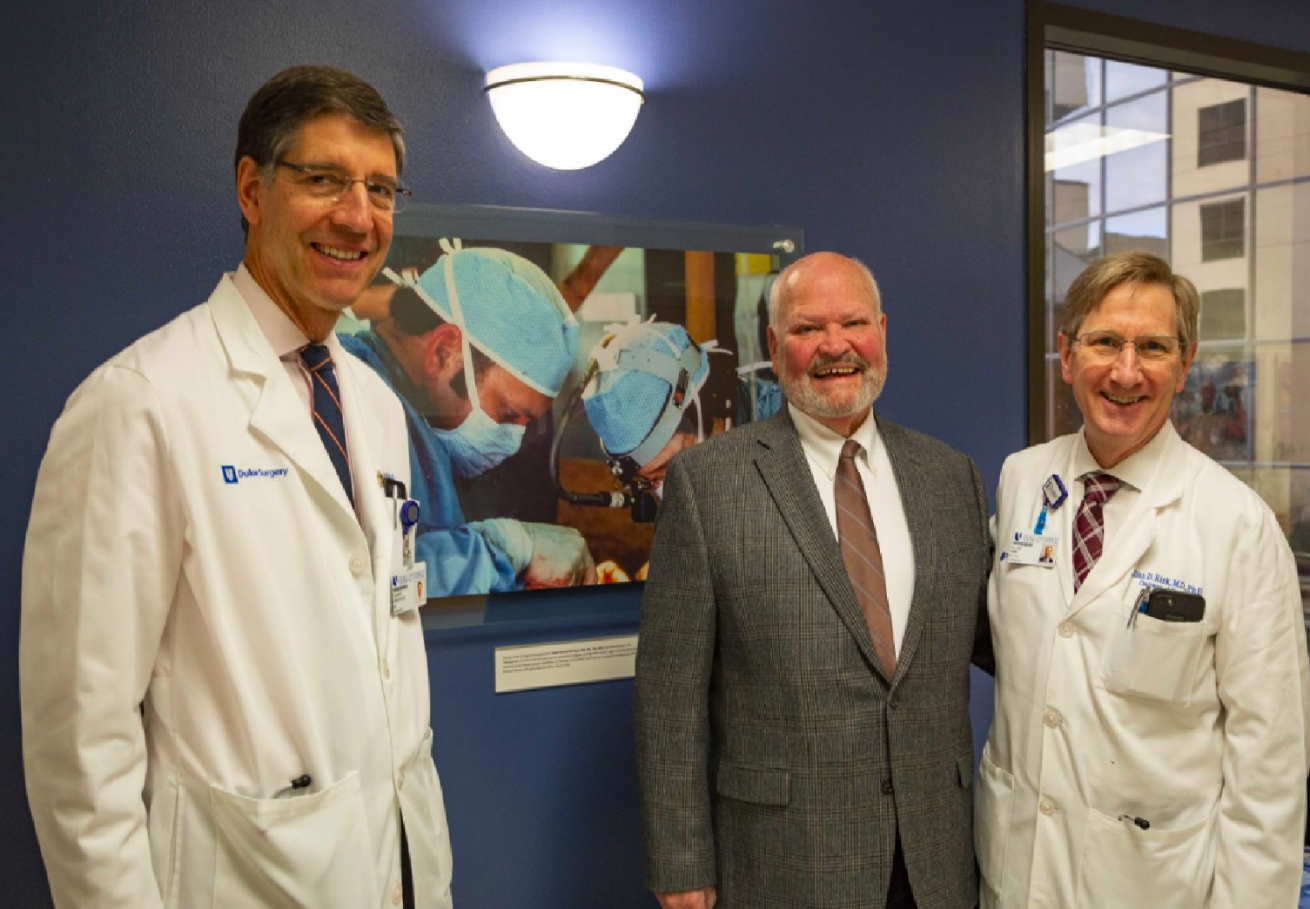
Randy Bollinger and Duke Surgery Department Chair Allan Kirk at the dedication of the Duke Organ Transplantation ward.

Randy won virtually every teaching award offered at Duke, including the David C. Sabiston, Jr. Teaching Award (1987), given to the top teacher in the department of surgery, the Golden Apple Award (1984 and 1989), given to the top teacher of medical students in any department, and the Distinguished Teacher Award (1989), given to the top teacher in the institution. If the impact of surgical educators is best measured by their trainees, then Randy Bollinger had a profound impact on the field of transplantation. His mentees that went on to careers in transplant surgery include Richard McCann, Ben Vernon, Stuart Knechtle, Allan Kirk, Bradley Collins, John Magee, Doug Farmer, Robert Harland, Rolf Barth, and Shu Lin. These were attracted to the field of transplantation by Randy's infectious enthusiasm, teaching skills, dedication to his patients, and consistent example of excellence. His lab jump-started the academic careers of many of these, including our own (4, 5), by creating an environment of inquiry, experimentation, opportunity, and collaboration. Randy was known to carry a small camera in his pocket, to use it frequently—long before the convenience of smart phones—and to document high points for his trainees or patients. Figure 2: Dr. Randy Bollinger (center) with Dr. Stuart Knechtle (left) and Dr. Allan Kirk (Surgery Department Chair, right) at dedication of the new Abdominal Transplant Unit, Duke Hospital). For his many contributions to his surgery department, he was recognized as a master surgeon, honored for embodying the ideals of an academic surgeon-clinician, teacher, and scientist. Forever dedicated to education, on his retirement from practice, he endowed a scholarship to support medical students interested in surgical research. The Bollinger Award has sponsored tuition for over 20 medical students, many of whom have gone on to pursue academic surgical practice.

An example of Dr. Bollinger's commitment to patient care was shown on the occasion of his 25th wedding anniversary. Although he had driven to a resort in Asheville, North Carolina, with his wife Monica to celebrate, there was a liver donor available that night, out of state. The Learjet that left Raleigh-Durham airport with a small team stopped in Asheville to pick up Randy who had driven to the airport with Monica who kissed him goodbye (with tears of love and regret). Not until years later did they find time to return to the same resort to actually celebrate. Monica was surely as flexible, patient, and forgiving as Randy was to his residents and students.
